# The Role of Mfsd2a in Nervous System Diseases

**DOI:** 10.3389/fnins.2021.730534

**Published:** 2021-09-10

**Authors:** Bei Huang, Xihong Li

**Affiliations:** ^1^Operational Management Office, West China Second University Hospital, Sichuan University, Chengdu, China; ^2^Key Laboratory of Birth Defects and Related Diseases of Women and Children (Sichuan University), Ministry of Education, Chengdu, China; ^3^Emergency Department, West China Second University Hospital, Sichuan University, Chengdu, China

**Keywords:** Mfsd2a, ICH, AD, SAE, MCPH, intracranial tumor, nervous system diseases

## Abstract

Major facilitator superfamily (MFS) is the maximum and most diversified membrane transporter, acting as uniporters, symporters and antiporters. MFS is considered to have a good development potential in the transport of drugs for the treatment of brain diseases. The major facilitator superfamily domain containing protein 2a (Mfsd2a) is a member of MFS. Mfsd2a-knockout mice have shown a marked decrease of docosahexaenoic acid (DHA) level in brain, exhibiting neuron loss, microcephaly and cognitive deficits, as DHA acts essentially in brain growth and integrity. Mfsd2a has attracted more and more attention in the study of nervous system diseases because of its critical role in maintaining the integrity of the blood-brain barrier (BBB) and transporting DHA, including inhibiting cell transport in central nervous system endothelial cells, alleviating BBB injury, avoiding BBB injury in cerebral hemorrhage model, acting as a carrier etc. Up to now, the clinical research of Mfsd2a in nervous system diseases is rare. This article reviewed the current research progress of Mfsd2a in nervous system diseases. It summarized the physiological functions of Mfsd2a in the occurrence and development of intracranial hemorrhage (ICH), Alzheimer’s disease (AD), sepsis-associated encephalopathy (SAE), autosomal recessive primary microcephaly (MCPH) and intracranial tumor, aiming to provide ideas for the basic research and clinical application of Mfsd2a.

## Introduction

As one of the two largest families of membrane transporters (Pao et al., 1998), the major facilitator superfamily (MFS) is the largest and most ubiquitously found family of secondary active membrane carriers (Saier et al., 2014; Finn et al., 2016). Its exporters have the ability to transport numerous substrates, ranging from small molecules such as organic and inorganic ions, to complex biomolecules including peptide and lipid moieties (Redhu et al., 2016). Research has shown that MFS transporters are associated with the occurrence and development of a variety of diseases. For example, lack of the Glucose transporter type 1 (GLUT1) transporter would lead to brain atrophy, developmental delay and other glucose deficiency disorders (Holman, 2020), neuronal lipofuscosis (Zare-Abdollahi et al., 2019), etc. Meanwhile, MFS is considered to have a good development potential in the transport of drugs for the treatment of brain diseases.

The major facilitator superfamily domain containing protein 2a (Mfsd2a) is an atypical solute carrier in MFS. In 2008, Mfsd2a was first identified as a new member of MFS by Angers et al. (2008). Mfsd2a could be expressed in liver, pons, corpus callosum, spinal cord, cerebellum and other tissues, especially in blood brain barrier (BBB) cerebral microvascular endothelial cells (Nguyen et al., 2014). Mfsd2a is essential for maintaining normal BBB (Zhao et al., 2020), and its protective effect on the BBB was obtained by inhibiting vesicular transcytosis (Qu et al., 2020). Mfsd2a-knockout mice showed obviously decreased levels of DHA in brain, exhibiting the loss of hippocampal and cerebellar neurons, severe anxiety, cognitive deficits, as well as microcephaly (Nguyen et al., 2014). More and more importance has been attached to the role of Mfsd2a in maintaining and regulating BBB function and its influence in nervous system diseases. Up to now, there have been reports about the role of Mfsd2a in cell fusion, placental development, cell cycle regulation and tumorigenesis. However, clinical research of Mfsd2a in nervous system diseases is rare. The regulatory effect of Mfsd2a deepens the understanding of the function of the BBB. It is probably conductive to effective drug delivery in the treatments of life-threatening infections in the central nervous system (CNS), neurodegenerative diseases and brain tumors (Wang et al., 2016). In this article, we reviewed the research of Mfsd2a in nervous system diseases, such as intracranial hemorrhage (ICH), Alzheimer’s disease (AD), sepsis associated encephalopathy (SAE), autosomal recessive primary microcephaly (MCPH), and intracranial tumor, aiming to provide a reference for the basic research and clinical application of Mfsd2a.

## Mfsd2a Gene and Protein Structure

Major facilitator superfamily is the maximum and most diverse membrane transporter (Pao et al., 1998), and acts as uniporters, symporters and antiporters, transporting materials inside and outside the cell (Reddy et al., 2012). MFS transporters include Mfsd1, Mfsd2a, Mfsd2b, and Mfsd3 etc. They could transport oligosaccharides, simple monosaccharides, drugs, enzyme cofactors, peptides, amino acids, vitamins, nucleobases, nucleosides, nucleotides, chromophores, organic, and inorganic anions cations, etc (Saier and Paulsen, 2001; Lorca et al., 2007; Chen et al., 2008; Yen et al., 2010).

Mfsd2a is an atypical solute carrier in MFS. In 2008, the study of Angers et al. (2008) suggested that Mfsd2a plays a part in adaptive thermogenesis. The *Mfsd2a* gene of mice was cloned for the first time and the human *Mfsd2a* gene was identified. The *Mfsd2a* genes are located on chromosome 4D2.2 in mice and on chromosome 1p33 in humans. The total length of *Mfsd2a* gene is about 14.3 kb, composed of 14 exons and 13 introns (Angers et al., 2008).

*Mfsd2a* gene encodes 530 amino acids in human. The asparagines at positions 217 and 227 are two N-conjugated glycosylation sites. Mfsd2a protein contains 12 α-helix fragments, each of which contains at least 17 amino acids (Quek et al., 2016). The sequence of Mfsd2a protein has little difference among different species, and has high consistency between mice and human. Transporters with different functions may have different folding patterns. Among varies transport families, MFS transporter superfamily has its unique folding mode, which is named “MFS fold” by researchers (Ethayathulla et al., 2014). Mfsd2a is a cell membrane protein, belonging to the secondary transporter MFS. It is highly expressed in many tissues, as well as in hunger-induced brown adipose tissue (BAT) and liver (Slocum et al., 2013). Besides, the study of Ben-Zvi et al. (2014) revealed that Mfsd2a was also expressed in the brain microvascular endothelial cells of BBB.

## Physiological Function of Mfsd2a

Researchers have identified how deficiencies in specific macromolecule transporters like Mfsd2a at the BBB could affect the integrity of overall cerebrovascular (Ben-Zvi et al., 2014; Nguyen et al., 2014). Mfsd2a maintains and regulates BBB, and also transports DHA. DHA acquisition in the brain is mediated mainly by the transporter Mfsd2a, which is expressed in BBB endothelial cells and many other cell types in the brain (Chan et al., 2018). Sterol regulatory-element binding protein (Srebp), identified as a transcription factor, is important for gene regulation that maintains cellular lipid homeostasis (Horton et al., 2002). As a physiological regulator of membrane phospholipid saturation, lysophosphatidylcholine (LPC)-DHA transported by Mfsd2a act in a feedback loop on Srebp activity during brain development (Chan et al., 2018).

### Mfsd2a Maintains and Regulates BBB

Approximately half of the dry weight of a mammalian brain comprises lipids, making it a lipid-rich organ in the body, second only to adipose tissue in lipid content (Betsholtz, 2015). BBB is critical in the establishment and maintenance of such lipid-rich organ (Keaney and Campbell, 2015). The permeability of BBB is altered to regulate the transport of substances in blood and brain (Hawkins et al., 2007). As a special non-permeable barrier in cerebral microvessels, BBB consists of blood vessels surrounded by astrocytic endfeet, endothelial cells united by tight junctions, pericytes embedded in vessel basement membranes (BMs), microglia and neurons (Dubois et al., 2014). The BBB forms the largest blood-brain exchange interface in adults (Nag and Begley, 2005). BBB restrains pathogens and other macromolecules from entering brain tissue through blood circulation (Kanda, 2013). It selectively transports nutrients needed by the brain and metabolic wastes, and prevents pathogenic substances, toxins and harmful macromolecules in the blood from entering and damaging brain tissue (Engelhardt and Liebner, 2014).

Blood-brain barrier breakdown has been proved to be associated with a variety of acute and chronic CNS disorders, implicating the potentially destructive effects of BBB disruption on brain function (Keaney and Campbell, 2015). The Mfsd2a is localized in cytoplasm and plasma membrane. Researchers evaluated the possible mechanism of BBB protection mediated by Mfsd2a through the cav-1/Nrf-2/HO-1 signaling pathway, finding that the overexpression of Mfsd2a alleviated brain edema and eliminated neurologic impairment resulting from surgical brain injury (SBI) while decreased Mfsd2a expression further worsened BBB functions and neurologic performance after SBI (Eser Ocak et al., 2020). The microenvironment rich in sphingosine-1-phosphate (S1P) in vascular endothelial extracellular matrix (ECM) dominates the formation and maintenance of BBB, while Mfsd2a is indispensable for brain endothelial cells outputting S1P. Mfsd2a and Spinster homolog 2 (Spns2) constitute a protein complex, ensuring the efficient transport of S1P (Wang et al., 2020). Andreone et al. (2017) found that lipids transported by Mfsd2a establish a unique lipid environment that restrains the formation of caveolae vesicle in CNS endothelial cells to inhibits transcytosis and ensure the integrity of BBB.

### Mfsd2a Transports DHA

Docosahexaenoic acid, a long chain polyunsaturated fatty acid, is mainly found in brain, nerve tissue and retina (Chan et al., 2018). A total of 60% of brain is composed of structural lipids, and the most abundant are docosahexaenoic acid (DHA) and arachidonic acid (AA). They act critically in brain growth and integrity (Basselin et al., 2012). DHA is proven essential to pre- and postnatal brain development (Kidd, 2007).

**FIGURE 1:**
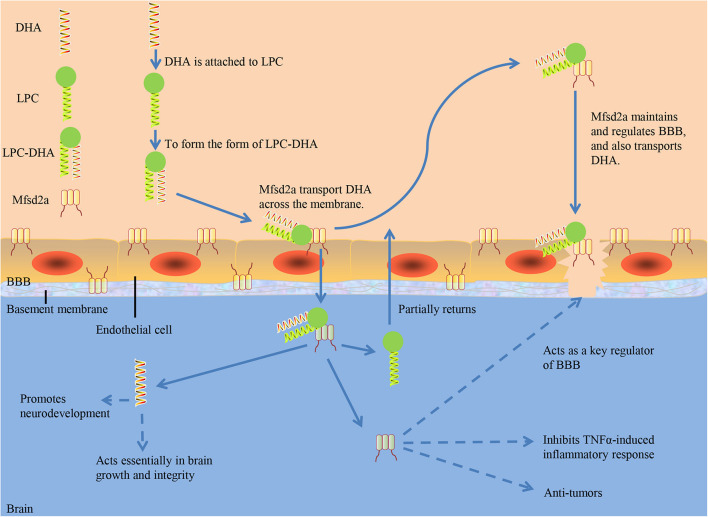
The role of Mfsd2a. DHA, docosahexaenoic acid; LPC, Lysophosphatidylcholine; BBB, blood-brain barrier; TNF-α, tumor necrosis factor-α.

Chan et al. (2018) demonstrated in mice that Mfsd2a is uniquely required for normal brain growth and DHA accumulation at postnatal BBB using vascular endothelial-specific and inducible vascular endothelial-specific deletion of Mfsd2a. Nguyen et al. (2014) found DHA content decreased significantly in the brain of Mfsd2a-knockout mice, suggesting that Mfsd2a is the main carrier for DHA to enter the brain through BBB. Serving as a sodium-dependent LPC symporter, Mfsd2a could be expressed at the BBB and transport LPCs containing DHA and other long-chain fatty acids (Wong et al., 2016). However, Mfsd2a could not transport non-esterified DHA and other non-esterified fatty acids. Only when DHA is attached to LPC to form the form of LPC-DHA, can Mfsd2a transport DHA across the membrane (Pauter et al., 2017; Figure 1). Dietary intake of LPC-DHA could increase DHA content significantly, improving brain function (Sugasini et al., 2017; Yalagala et al., 2019; Scheinman et al., 2021). Brain intake of DHA from free DHA was lower than that from LPC-DHA (Chen et al., 2015; Subbaiah et al., 2016). Increasing DHA in the brain through diet is dependent on DHA-LPC production, and DHA from LPC enriches the brain and acts effectively (Sugasini et al., 2019). Common plasma-derived LPCs with long-chain fatty acids, including LPC palmitate and LPC oleate, can be transported by Mfsd2a, but LPCs carrying less than 14 carbonyl chains cannot (Nguyen et al., 2014). The transport of LPC through Mfsd2a has been proved to be indispensible for human brain growth. Dietary supplementation with specific form of DHA is a novel treatment for reducing the risk of neurodegenerative disorders (Balakrishnan et al., 2021). Researchers presented three-dimensional structure models of human Mfsd2a derived from homology modeling. They recognized Lys-436 as a pivotal residue for transport, and also demonstrated that the interaction between a negatively charged headgroup and Lys-436 is necessary for transport. Then a novel transport mechanism was proposed, in which LPCs are “flipped” within the transporter cavity by rotating around Lys-436, resulting in net transport from the outer to the inner leaflet of the plasma membrane (Quek et al., 2016; Table 1).

**TABLE 1:** The main physiological functions of Mfsd2a in nervous system.

First author, year	Function	Mechanism	Species
Ben-Zvi et al., 2014	To act as an essential regulator of BBB function	It might work via inhibiting cell transport in CNS endothelial cells	Mice
Andreone et al., 2017	To maintain and regulate the integrity and permeability of BBB	Specifically inhibits vesicle mediated transcytosis in BBB capillary endothelial cells	Mice
Yang et al., 2017	To avoid BBB injury in cerebral hemorrhage model	Mfsd2a overexpresses in perihematomas	Mice
Qu et al., 2020	To promotes brain development and the formation of cognitive abilities	Mfsd2a could alleviate BBB injury and improve cognitive function in CCH rats	Rats
Quek et al., 2016	To act as a carrier, transporting DHA into the brain	Lys-436 was identified as a key residue for transport	Mice

*BBB, blood-brain barrier; DHA, docosahexaenoic acid; CNS, central nervous system; CCH, chronic cerebral hypoperfusion.*

## The Study of Mfsd2a in Nervous System Diseases

The injury of BBB is related to the pathogenesis of various acute and chronic nervous system diseases. Reducing the permeability of BBB could reduce the mortality related to brain injury. Mfsd2a is involved in nervous system diseases by regulating BBB integrity.

### Intracranial Hemorrhage

Intracranial hemorrhage, a subtype of stroke, is associated with a high mortality rate. Survivors often have severe nerve injury. Accounting for 20–30% of all strokes in Asia and 10–15% of strokes in the Europe, United States, and Australia, ICH has approximately two million cases worldwide each year (Keep et al., 2012). ICH is still the deadliest and most difficult type of stroke to treat (Kase, 1995). So far, the treatment options of ICH are limited, and only supportive care and rehabilitation could improve it (Anderson, 2009; Chuang et al., 2009).

Vascular interruption is the root cause of cerebral hemorrhage, including intracerebral hemorrhage, subarachnoid hemorrhage and intraventricular hemorrhage. The etiology of the disease also includes the damage of BBB caused by cerebral hemorrhage, which is an important part of brain injury after cerebral hemorrhage. There is growing evidence that the secondary effects of ICH include cerebral edema, inflammation autophagy, BBB disruption, as well as cellular apoptosis and necrosis (Lee et al., 1997; Xi et al., 2006; Lapchak and Araujo, 2007; Ducruet et al., 2009; Selim, 2009). In ICH and subarachnoid hemorrhage (SAH) rats, the expression of Mfsd2a in the brain tissue around the hematoma significantly decreased, which may contribute to the destruction of BBB (Zhao et al., 2020). After ICH, Mfsd2a-knockout mice showed obvious increases in BBB permeability, neurological impairment score, and brain water contents, which were rescued by overexpression of Mfsd2a around the hematoma (Yang et al., 2017). Meanwhile, the results suggested that the protective effect of Mfsd2a against BBB injury may be achieved by inhibiting vesicular transcytosis after ICH. Overexpression of Mfsd2a inhibited the vesicle-mediated transcytosis, reduced the number of vesicles, significantly reduced the hematoma level of brain tissue in ICH mice, and weakened BBB injury caused by ICH. It suggested that Mfsd2a might be used to prevent BBB injury by inhibiting the transcytosis after ICH.

### Alzheimer’s Disease

Characterized by low DHA levels in blood lipids, AD is a neurodegenerative disease. BBB dysfunction and/or breakdown occurs in AD prior to dementia, brain atrophy and/or neurodegeneration (Montagne et al., 2017). Using novel AD mice with chronic cerebral hypoperfusion (CCH) model, Shang et al. (2019) studied the expression changes of two main amyloid-β transport receptors, namely receptor for advanced glycation end products (RAGE) and low-density lipoprotein receptor related protein-1 (LRP1). They found that CCH decreased the expression of Mfsd2a. In the study of Qu et al. (2020), a CCH rat model was built through making permanent bilateral common carotid artery occlusion (2VO) in rats. Following the 2VO treatment, rats had cognitive impairment, increased BBB leakage in hippocampus, and decreased Mfsd2a protein expression. Mfsd2a overexpression within the hippocampus reversed those changes. These results suggest that Mfsd2a could alleviate BBB injury and improve cognitive function in CCH rats.

The increase of age is related to increasing low-grade chronic inflammation/inflammaging, probably leading to the neurodegenerative process of AD (Onyango et al., 2021). Researchers found that long-term supplementation of fish oil (FO) is conducive to improving AD pathology, and even short-term intake of FO showed improvement in phospholipid composition. After FO supplementation, the expression of Mfs2a, an omega-3 transporter, did not change however. Besides, the application of FO in early stages of AD pathology markedly affected the plaque burden in 5xFAD brains (Milanovic et al., 2018). N-6 polyunsaturated fatty acids (N-6 PUFA) and their derived molecules, such as lipid mediators derived from arachidonic and linoleic acid, are pro-inflammatory in the peripheral region. The number of astrocytes was positively correlated with the expression of Mfs2a in the brain (Tiwary et al., 2018). Alashmali et al. (2019) found that the upregulation of Mfsd2a in mice adequately fed with N-6 PUFA is possibly due to the high presence of astrocytes, maintaining Mfsd2a expression in the brain and controlling BBB homeostasis. In the future, studies on Mfsd2a-knockout mice are expected to clarify the mechanism by which N-6 PUFA affects the expression of *Mfsd2a* in the brain.

There is a significant difference in the expression of Mfsd2a protein in brain between patients with AD and normal human, which may be the key to understand the role of Mfsd2a in the pathological process of AD. Sánchez-Campillo et al. (2019) first analyzed Mfsd2a carrier in patients with AD, indicating that the Mfsd2a level in the whole blood might be a potential biomarker for the disease.

### Sepsis-Associated Encephalopathy

Sepsis is a potentially life-threatening disease, resulting from the dysregulated host systemic response to infection (Atterton et al., 2020). SAE is a common complication of sepsis and has a high risk of death, and can occur at any stage of sepsis. Encephalopathy increases mortality rate in septic patients (Zhang et al., 2012).

Acute infection worsens existing chronic diseases or leads to new ones, resulting in poor long-term prognosis of survivors of acute diseases (Mayr et al., 2014). Mfsd2a is expressed in BBB capillary endothelial cells. It could transport LPC, which connects long chain fatty acids, into the brain microvascular endothelial cells. It is an important supporter to maintain the integrity and function of BBB (Nguyen et al., 2014). When BBB is damaged, its permeability is enhanced. Endotoxin and inflammatory factors enter the brain tissue, leading to impaired or even loss of brain function (Hwang and Kim, 2014). Ben-Zvi et al. (2014) found that Mfsd2a was selectively expressed in blood vessels containing BBB in the CNS, and its expression on endothelia was regulated by pericytes to maintain the integrity of BBB, identifying Mfsd2a as a key BBB function regulator which might work by suppressing endothelial transcytosis in the CNS.

He et al. (2018) hypothesized that in the development of sepsis, intraperitoneal hypertension (IAH) was usually accompanied by various degrees of IAH, internal jugular vein pressure was increased by intra-abdominal pressure, and pressure in BBB capillary lumen was also increased. Endothelial cells then felt these changes in pressure, causing the expression, distribution and function of BBB permeability related protein abnormal at first. For example, Mfsd2a made the permeability of BBB increase (He et al., 2018). Therefore, large amounts of water, various cytokines including pro-inflammatory cytokines, pseudo-neurotransmitters, etc. entered the brain parenchyma, resulting in brain damage and then triggered SAE. The gap junction was broken due to the severe damage of the structural protein, when the abdominal pressure rised to a certain level and lasted for a period of time. Lots of neurotoxic substances poured into the brain thereby, causing the rapid deterioration of SAE. Controlled and prospective clinical trials in SAE patients are urgently needed to enrich the experience of evidence-based medicine intervention (Chung et al., 2020).

In addition, tumor necrosis factor-α (TNF-α) could decrease the expression of Mfsd2a. The decreased expression of Mfsd2a could inhibit TNF-α-induced inflammatory response and alleviate intestinal inflammation, suggesting that Mfsd2a can regulate the response of intestinal endothelial cells to inflammation (Ungaro et al., 2017).

### Microcephaly

Autosomal recessive primary microcephaly is a neurodevelopmental disorder falling into two forms: primary MCPH at birth and secondary MCPH after birth (Cowie, 1960). Secondary MCPH is concerning progressive neurodegenerative disease compared with primary MCPH, a prenatal developmental neurogenic disorder (Naveed et al., 2018). Patients with MCPH may or may not have mild to severe intellectual disability, and may also have short stature, seizures or congenital hearing loss (Darvish et al., 2010). The head size of MCPH patients is less than 3–4 standard deviations, and the area of cerebral cortex is reduced (Araujo et al., 2014). Scala et al. (2020) analyzed 27 cases of MCPH, showing that the low expression of Mfsd2a was related to the pathological manifestations of MCPH. So far, 18 loci of MCPH have been detected, and Mfsd2a is one of them, so Mfsd2a is also called MCPH15 (Naveed et al., 2018).

Selectively expressed in human microvascular endothelial cells, Mfsd2a is the main DHA transporter in the brain (Zhou et al., 2019). Brain requires DHA and LPC, which are essential for neurodevelopment, through Mfsd2a. The expression of Mfsd2a in basal plasma membrane of umbilical vein was positively correlated with the amount of DHA-LPC (Ferchaud-Roucher et al., 2019). Mutations of Mfsd2a malfunction its activity in brain endothelial cells, resulting in MCPH. Changes in maternal blood levels of Mfsd2a during pregnancy may affect placental nutrient transport and fetal neural development. Mfsd2a expression is low in gestational diabetes mellitus (GDM) placentas. This affects maternal-fetal transport of DHA. People with homozygous inactivation mutations of *Mfsd2a* gene have severe MCPH and intellectual impairments (Sánchez-Campillo et al., 2020). These suggest that Mfsd2a expression level during pregnancy may be a potential biomarker for early diagnosis of neurodevelopmental abnormalities in children. The study of Toufaily et al. (2013) showed that forskolin-induced BeWo cell fusion was first paralleled to the increased Mfsd2a expression; Down-regulated expression of Mfsd2a confirmed its key role in BeWo fusion; and Mfsd2a expression decreased in placentas of severe preeclampsia. All the results indicated the critical role of Mfsd2a in both trophoblast fusion and placental development.

According to the genotype-phenotype data of an 11-year-old male patient with autosomal recessive primary MCPH15, Razmara et al. (2020) found that p(Val81del) may be a pathogenic variant causing nonfatal MCPH, but further molecular methods and research data are needed to identify the genotype-phenotypic association of Mfsd2a accurately. Modification of genetic factors or nutritional supplementation has the potential to regulate the severity of disease, and the treatment options for affected individuals should be considered (Harel et al., 2018). Guemez-Gamboa et al. (2015) found that 4 children showed severe intellectual disability and motor impairment, and died in childhood. The functional analysis of Mfsd2a mutations showed that the transport function of Mfsd2a carrying both DHA and other long-chain fatty acids linked to LPC was lost. Alakbarzade et al. (2015) investigated 10 MCPH patients of Pakistani origin. They had homozygous mutations in Mfsd2a, which was not completely inactivated. The Mfsd2a retained some ability to transport LPC- long-chain aliphatic acyl chains. These patients presented with MCPH milder than the previous cases. These two studies reflected the association between the damage degree to Mfsd2a protein caused by mutation, losing function totally or partially, and the symptom severity of MCPH.

### Intracranial Tumor

Intracranial tumors include primary brain tumors and brain metastases (BMs). The best treatment for them is surgical resection. However, it is extremely difficult to completely remove the tumor in a large area, due to the unresectable nature of normal brain tissue and the extensive and invasive growth of malignant tumor in the brain.

Tiwary et al. (2018) treated human brain microvascular endothelial cells (HBMECs) with different cytokines known to affect the properties of endothelial barrier, including TGFβ1, bFGF/FGF2 and VEGF, finding that both TGFβ1 and bFGF induced Mfsd2a expression, whereas VEGF inhibited the expression of *Mfsd2a* gene in HBMECs. The decreased expression of Mfsd2a in patients with BMs may be related to the lack of TGFβ1 and bFGF signaling pathways in pathological conditions. The recovery of DHA metabolism in patients with brain tumor is probably a new therapeutic strategy to prevent the survival and growth of metastatic tumor cells.

The study of Moritake et al. (2017) revealed that Mfsd2a acted critically in unfolded protein response to exposure to tunicamycin (TM). The lack of Mfsd2a expression in cells indicated TM resistance, while cells with high Mfsd2a expression showed high sensitivity. Mfsd2a is a recognized transporter of TM, which enters the body across cell membrane. It also could promote transporting TM into tumor cells (Bassik and Kampmann, 2011). Whether Mfsd2a is the receptor of other anti-tumor antibiotics remains to be confirmed. Some researchers believe that *Mfsd2a* gene is a tumor suppressor which modulates extracellular matrix attachment and cell cycle (Spinola et al., 2010), and is essential for CD8+ T lymphocytes to function (Piccirillo et al., 2019). Mfsd2a has been confirmed to enhance transmembrane transport in vascular endothelial cells by reducing pericyte density in mice. Studies also found that Mfsd2a expression in brain endothelial cells depends on the presence of astrocytes. However, the pathogenic mechanism and prognostic monitoring value of Mfsd2a in intracranial tumors need to be further studied.

In addition, the expression of Mfsd2a decreased in patients with brain metastases. Therefore, the transport function of LPC-fatty acid is impaired and cerebral vascular endothelium is damaged, which leads to BBB dysfunction (Tiwary et al., 2018).

Tumor necrosis factor-α (TNF-α) could decease the expression of Mfsd2a. Decease of Mfsd2a expression can inhibit TNFα-induced inflammatory response and alleviate intestinal inflammation, suggesting that Mfsd2a can regulate the response of intestinal endothelial cell to inflammation (Ungaro et al., 2017). Mfsd2a expression is downregulated in hepatocellular carcinoma (HCC), and the expression levels of Mfsd2a can be an independent prognostic indicator in HCC patients (Xing et al., 2019).

The study of Xi et al. (Shi et al., 2018) suggested that Mfsd2a might have an effect on angiogenesis and inhibit the occurrence and development of gastric cancer. *Mfsd2a* is also a suppressor gene in lung cancer because its exogenous expression blocked the G1 phase of cell cycle, impaired adhesion and migration properties, thereby affecting tumor growth and development via controlling cell cycle, cell motility, and matrix attachment (Spinola et al., 2010). However, data on the role of Mfsd2a in tumors is limited. It is still controversial (Table 2).

**TABLE 2:** The role of Mfsd2a in nervous system related diseases.

First author, year	Diseases	Expression of Mfsd2a	The role of Mfsd2a	Species
Yang et al., 2017	ICH	Decreased	It alleviates BBB injury and neurological dysfunction	Mice
Sánchez-Campillo et al., 2019	AD	Decreased	It may be a potential biomarker for AD process	Human
He et al., 2018	SAE	Abnormal	It made the permeability of BBB increase and thus large amounts of water and various cytokines entered the brain parenchyma, leading to brain damage, and then SAE	Mice
Alakbarzade et al., 2015; Guemez-Gamboa et al., 2015; Sánchez-Campillo et al., 2020; Scala et al., 2020	MCPH	Decreased	Mutations in Mfsd2a malregulate the activity of transporters in cerebral endothelial cells, leading to MCPH	Human
Xing et al., 2019	BM	Decreased	It is associated with the TGFβ1 and bFGF pathways	Mice

*BBB, blood-brain barrier; ICH, intracranial hemorrhage; AD, Alzheimer’s disease; SAE, sepsis-associated encephalopathy; MCPH, microcephaly; BM, brain metastases.*

## Discussion

An intact BBB severely restricts drug delivery to the CNS (Ben-Zvi et al., 2014), while Mfsd2a plays a key role in BBB permeability regulation and integrity maintenance by specifically inhibiting vesicle mediated transcytosis in BBB capillary endothelial cells, so the targeting of Mfsd2a might help promote effective drug delivery in the treatment of life-threatening CNS infections, neurodegenerative diseases and brain tumors. Mfsd2a restrains transport of most molecules via transcytosis and also enhances specific transport of LPC-coupled derivatives such as LPC-DHA (Wang et al., 2016). Such dual function of Mfsd2a proposes two pharmacological strategies for delivering drugs through BBB based on Mfsd2a. On the one hand, inhibiting Mfsd2a has the potential to open up BBB for drug delivery across the BBB. Therefore, the combination of Mfsd2a inhibitors with macromolecular drugs such as recombinant proteins, antibodies may be a method of allowing drugs to cross the BBB and accumulate in the corresponding sites. In this case, the development of Mfsd2a inhibitors is critical, but the current discovery of it is very limited. On the other hand, Mfsd2a transports some LPC-coupled derivatives through BBB, so the development of drugs chemically coupled with LPC is also a therapeutic strategy. As a drug carrier, LPC may transport LPC drug complex to the CNS by Mfsd2a to achieve therapeutic purposes.

## Conclusion and Prospects

Mfsd2a plays an important role in maintaining and regulating BBB, promoting DHA transport, brain development, and cognitive formation. At present, the research of Mfsd2a in nervous system diseases is limited, and there are many problems need to be further explored. What is the relationship between Mfsd2a and pericytes and glial cells in maintaining and regulating BBB integrity and permeability? Does Mfsd2a play a role in transport directly or indirectly? Are there other regulatory factors? Does Mfsd2a have its unique structural characteristics and transport mechanism? What is the relationship between the role of Mfsd2a in promoting brain development, cognition and memory formation and the pathogenesis and prognosis of neurological diseases such as ICH, AD, SAE, MCPH, and intracranial tumor? Mfsd2a may protect BBB by inhibiting vesicle endocytosis after intracerebral hemorrhage. The difference of Mfsd2a protein expression between AD patients and normal human brain may be the key to study the role of Mfsd2a in the pathological process of AD. Although the incidence rate and clinical relevance of SAE are high, the pathological mechanism of its acute and chronic stages is not yet fully understood. Establishing effective biomarkers is of great significance. The degree of Mfsd2a protein damage caused by the mutation and its correlation with the severity of MCPH need to be further clarified. The pathogenic mechanism and prognostic monitoring value of Mfsd2a in intracranial tumors are expected to be further studied.

## Author Contributions

BH drafted the manuscript and prepared the tables. XL edited and revised the manuscript. BH and XL approved final version of the manuscript. Both authors contributed to the article and approved the submitted version.

## Conflict of Interest

The authors declare that the research was conducted in the absence of any commercial or financial relationships that could be construed as a potential conflict of interest.

## Publisher’s Note

All claims expressed in this article are solely those of the authors and do not necessarily represent those of their affiliated organizations, or those of the publisher, the editors and the reviewers. Any product that may be evaluated in this article, or claim that may be made by its manufacturer, is not guaranteed or endorsed by the publisher.
